# Electrospun nanofibers for gastric cancer: local therapeutics, biomimetic models, and diagnostic interfaces

**DOI:** 10.3389/fonc.2026.1795748

**Published:** 2026-04-15

**Authors:** Zhao Wang, Yuanyuan Liu, Yihui Li, Jiangong Wang

**Affiliations:** 1 Department of General Surgery, Tangshan People’s Hospital, Tangshan, China; 2 The Third Department of Cancer Radiotherapy and Chemotherapy, Affiliated Hospital of North China University of Science and Technology, Tangshan, China; 3 Department of Radiotherapy and Chemotherapy VIII, Tangshan People’s Hospital, Tangshan, China

**Keywords:** drug delivery, electrospun, gastric cancer, nanofibers, peritoneal metastasis

## Abstract

Electrospun nanofibers have emerged as a versatile local-regional platform for gastric cancer, particularly for postoperative recurrence control and peritoneal metastasis, where systemic therapies often fail to achieve sufficient and durable intraperitoneal exposure. Owing to their high surface area, interconnected porosity, and engineerable architecture, electrospun membranes enable programmable local pharmacokinetics and tunable cell–material interactions by regulating fiber diameter, pore connectivity, and alignment. Advanced designs, including coaxial, Janus, multilayer, gradient, and porous fibers, further expand the therapeutic window by separating drug compatibility from release behavior and supporting sequential or compartmentalized delivery. In perioperative settings, electrospun patches can function as locoregional depots to suppress microscopic residual disease while simultaneously facilitating infection control and tissue repair. For peritoneal metastasis, electrospun membranes can sustain intraperitoneal drug exposure and incorporate microenvironment-responsive sensitization strategies targeting hypoxia, redox adaptation, and ferroptosis resistance. Beyond therapy, electrospun scaffolds provide biomimetic gastric cancer models that better recapitulate diffusion barriers, metabolic gradients, and mechanobiological cues, improving the translational relevance of drug-response evaluation. Nanofiber-based interfaces also support longitudinal monitoring by enhancing the capture and analysis of circulating tumor cells and other low-abundance biomarkers. Finally, we highlight key translational considerations, including quality-by-design, critical quality attributes, sterilization robustness, and scalable manufacturing, to accelerate clinical adoption of electrospun products for gastric cancer management.

## 1 Introduction

Electrospinning has matured from a laboratory curiosity into a versatile nanofiber manufacturing platform that enables precise control over fiber diameter, porosity, alignment, composition, and hierarchical architectures, thereby offering an unusually broad design space for biomedical translation (1–5). The physical foundation of the process—electrically driven jet formation and thinning from a Taylor cone—has been established for decades (6–8), while contemporary innovations have expanded the repertoire from simple polymer fibers to multifunctional membranes that integrate controlled release, catalytic/photothermal components, and bioactive interfaces (9–11). Within biomedicine, electrospun nanofibers are particularly attractive because they combine extracellular-matrix-like fibrous topology with high surface area and tunable degradation, supporting both drug delivery and tissue-interactive functions (11, 12). In cancer research, these attributes have catalyzed rapid growth in nanofiber-enabled strategies spanning localized therapy, implantable drug depots, 3D tumor modeling, and biosensing—use-cases where conventional nanoparticles or bulk hydrogels can face constraints in mechanical integrity, spatial controllability, or long-term site retention (13–16).

Gastric cancer remains a major global health burden, characterized by substantial geographic heterogeneity, frequent late-stage diagnosis, and high mortality driven by metastatic spread and therapy resistance (17–19). Although systemic therapy has advanced-perioperative multi-agent chemotherapy improving outcomes in resectable disease (20), and biomarker-directed regimens reshaping advanced-stage care through HER2 targeting, immune checkpoint inhibition, and newer antibody strategies (19, 21–24)—several clinical bottlenecks persist. Tumor heterogeneity, variable immunogenicity, treatment-limiting toxicities, and sanctuary sites contribute to suboptimal durable control in a large proportion of patients (18).

In particular, peritoneal metastasis is common and clinically devastating. In colorectal cancer, peritoneal metastases are detected in 5–10% of patients undergoing resection (25). Registry-based studies further report metachronous peritoneal metastasis in 3.5% of patients by 18 months, increasing to 6% within 5 years, with higher rates in selected high-risk subgroups (26). In gastric cancer, recent population-based studies report synchronous peritoneal metastases at primary diagnosis up to 21% (26). Drug delivery to peritoneal implants is challenged by rapid clearance, uneven exposure, and cumulative systemic toxicity when intensifying systemic regimens. Even locoregional approaches such as cytoreductive surgery plus HIPEC remain controversial, with efficacy signals varying across studies and patient subsets and with procedural morbidity and standardization issues complicating broad adoption (27).

These gaps align closely with the core strengths of electrospun nanofiber systems: (i) sustained, spatially localized drug release; (ii) conformal coverage of complex anatomical surfaces; (iii) programmable multicomponent delivery (chemo plus microenvironment modulation); and (iv) scaffold functions that can support tumor modeling and sensing (28–31). Recent gastric-cancer-relevant examples illustrate this promise. Drug-loaded nanofiber sheets have demonstrated prolonged antitumor activity in gastrointestinal cancer xenograft settings and peritoneal dissemination models, leveraging sustained release from implantable membranes to improve local exposure while potentially reducing systemic peaks (32). More complex membranes designed for intraperitoneal therapy in advanced gastric cancer with peritoneal metastases have integrated continuous chemotherapy release with microenvironment-responsive mechanisms to intensify local cytotoxicity *in vivo*, highlighting the feasibility of “therapeutic membranes” rather than freely circulating carriers (33, 34). Beyond therapy, electrospun scaffolds have been used to construct three-dimensional gastric cancer culture systems for chemosensitivity testing, supporting the broader trend toward biomimetic tumor models that may better recapitulate drug penetration, cell–matrix interactions, and treatment response than conventional two-dimensional monolayers (35). Finally, electrospun nanofibers are increasingly deployed as high-performance transducer interfaces in biosensors, where their porosity and surface chemistry facilitate immobilization of recognition elements and enhance analyte access—an enabling capability for gastric-cancer-relevant biomarker workflows in liquid biopsy and point-of-care diagnostics (36).

Against this backdrop, an “electrospun nanofibers and gastric cancer” lens is timely: it connects a rapidly innovating materials platform to clear unmet clinical needs in locoregional control, precision modeling for therapy selection, and biomarker-driven monitoring (37–40). In this review, we synthesize progress in electrospinning fundamentals and advanced nanofiber architectures; map materials/design choices to gastric-cancer-specific therapeutic and diagnostic objectives; and critically evaluate translational barriers. We further propose design principles and study standards that could accelerate rigorous preclinical-to-clinical progression of nanofiber-based interventions in gastric cancer (16, 19, 41).

## 2 Electrospinning principles and design space

Electrospinning is well suited for gastric cancer research and locoregional therapy not merely because it produces nanofibers, but because it enables structural engineering. A standard setup—syringe pump, spinneret, high-voltage source, and collector—drives a stable charged jet and deposits fibers into membranes. This expands the design space beyond formulation. Fiber diameter, pore connectivity, and alignment become tunable variables that shape local transport, drug exposure, and cell–material interactions (**Table 1**).

**Table 1:** Electrospinning architectures and implications for gastric cancer applications.

Architecture	Payload localization	Release profile	GC-relevant use-cases	Representative refs
Single-nozzle (blend)	Matrix-dispersed	Burst + diffusion/degradation	Postoperative patch; anti-adhesion barrier + drug	(42, 43)
Coaxial (core–shell)	Core protected; shell functional	Two-phase; reduced burst; staged	Cytotoxic + sustained anti-metastasis; protect biologics	(44, 45)
Emulsion electrospinning	Droplet-derived compartments	Sustained; tunable	Encapsulate hydrophilic drugs within hydrophobic fibers	(45, 46)
Side-by-side (Janus)	Two chemistries exposed	Parallel/asymmetric release	Dual-function surfaces (anti-adhesion + bioactive)	(47, 48)
Multilayer/stacked meshes	Layer-defined compartments	Programmable, multi-stage	Sequential therapy (debulking → suppression)	(49, 50)

### 2.1 Process–structure–property mapping

**Figure 1:**
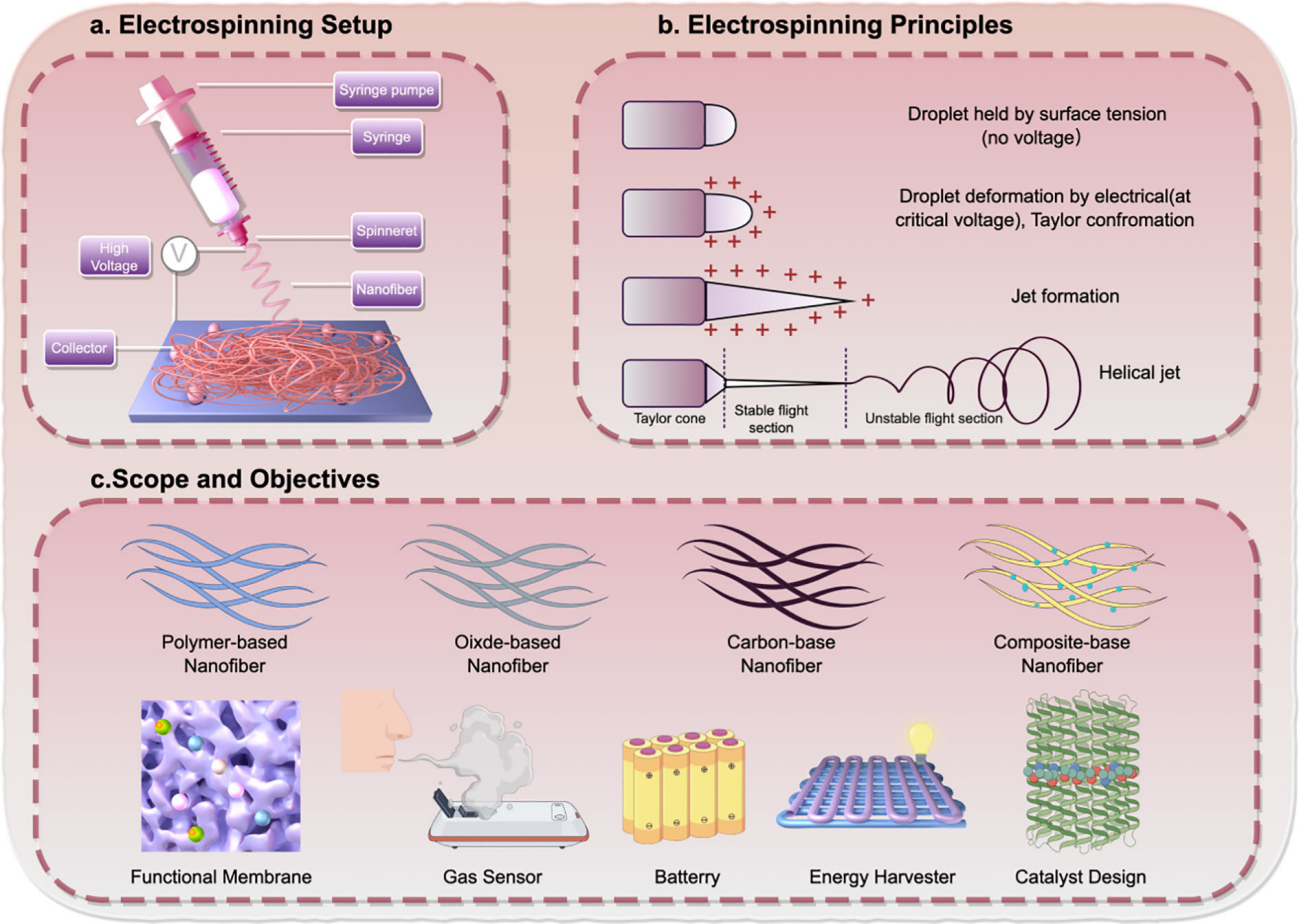
Schematic illustration of an electrospinning setup, fiber-forming mechanism, and the structural design space of electrospun nanofibers. **(a)** A representative electrospinning system comprising a syringe pump and syringe, a spinneret, a high-voltage power supply, and a collector. Under an applied electric field, the polymer solution is drawn into a charged jet and deposited to form a nanofibrous membrane. **(b)** Key steps of jet initiation and fiber formation. The pendant droplet is stabilized by surface tension until a critical voltage induces electrohydrodynamic deformation and the emergence of a Taylor cone. A jet is then emitted, undergoing a straight (stable) segment followed by a whipping (unstable) regime, before solidifying into a continuous nanofiber network on the collector. **(c)** Overview of major electrospun nanofiber material classes and functional extensions, including polymer-based, oxide-based, carbon-based, and composite nanofibers. These architectures can be further engineered into functional membranes, sensing interfaces, energy-related devices, and catalytic platforms, providing a general framework for structure-driven design in biomedical applications.

Electrospinning is a fiber‐forming process in which a polymer solution is electrically charged and continuously stretched under a high-voltage electrostatic field, followed by solvent evaporation to generate nanoscale fibers (51). The typical electrospinning setup mainly consists of a high-voltage power supply, a syringe pump, and a grounded collector. During operation, the pendant droplet at the needle tip is governed by two competing forces: surface tension that stabilizes the droplet shape and electrostatic forces that deform it (52, 53). As the electric field strengthens, the droplet elongates into a conical meniscus known as the Taylor cone (54). Once the electrostatic force exceeds the surface tension, a charged jet is ejected from the cone apex and accelerates toward the collector. Along its flight path, the jet undergoes further stretching and thinning, while the solvent continuously evaporates. Eventually, the solidified jet deposits on the collector to form a nonwoven nanofibrous mat (55). Importantly, the final fiber morphology is highly sensitive to multiple controllable parameters, which are commonly grouped into solution factors, processing factors, and environmental conditions (56) (**Figure 1**).

Anchored to the clinical realities of gastric cancer, this process–structure mapping ultimately converges on two use cases. For peritoneal metastasis, the priority is that the material can remain in the peritoneal cavity, release drugs in a stable manner, and sensitize tumors locally. Accordingly, electrospun composite systems are often engineered to sustain an effective exposure window for agents such as paclitaxel, while exploiting the acidic and reducing peritoneal microenvironment to trigger local reactions that amplify cytotoxicity. This design couples drug pharmacokinetics and microenvironmental sensitization within a single membrane (57). In contrast, peri-anastomotic management after gastrectomy is not defined by maximal tumor killing. It is defined by uneventful healing, an intact barrier, and controlled adhesions (58). A representative example is a biodegradable electrospun membrane loaded with ginsenoside Rg3, which reduced adhesion severity in an intraperitoneal adhesion model and was accompanied by decreased inflammatory and oxidative-stress markers (59). Together, these findings indicate that electrospun materials can function not only as drug depots, but also as local microenvironment modulators to mitigate perioperative complications (60).

Fiber alignment carries a particularly sensitive implication in gastric cancer–oriented research. It does not only affect whether cells “grow well”; it can directly reprogram where tumor cells migrate and how fast they disseminate (61). Aligned fibers create ECM-like linear tracks that strengthen directional motility. Mechanistically, this effect is sustained by a Caveolin-1–linked program involving integrin β1 endocytosis, cytoskeletal tension remodeling, and YAP activation (62, 63). Thus, “alignment promotes migration” is not a hypothesis. It is a mechanistically grounded outcome. Importantly, cells in 3D do not respond only to perfectly ordered cues. They can also integrate noisy contact-guidance signals and adopt a polarized, migratory state. This implies that small changes in alignment can produce nonlinear shifts in invasive behavior (64). Aligned architectures are therefore well suited for modeling invasion and metastatic traits, but they should be used with caution in postoperative anti-dissemination barriers, where excessive alignment may unintentionally facilitate migration. Consistent with this view, CAV1-driven integrin trafficking and fibronectin turnover have been shown to shape matrix remodeling and migration strength, providing actionable entry points for combining alignment control with interface engineering (65).

Finally, electrospun scaffolds can reshape drug-response readouts. In gastric cancer, cells cultured in electrospun 3D matrices often require higher drug doses to achieve killing comparable to 2D monolayers. This highlights a practical pitfall: 2D systems tend to overestimate efficacy and underestimate tolerance, whereas electrospun biomimetic models better reflect therapeutic pressure *in vivo*.

### 2.2 Multicomponent electrospun architectures

Electrospinning is recognized as a highly efficient and well-established technique for fabricating nanofibrous scaffolds, yielding fibers with diameters spanning the nanometer to micrometer range (66). The mechanical properties and surface topographical features of these scaffolds significantly influence cell migration, cancer progression, metastatic and invasive potential, while also modulating tumor cell drug resistance and gene expression.

Owing to its unique capability to produce nanofibers that closely mimic both the structural and biochemical characteristics of the gastric tumor microenvironment, electrospinning has emerged as a vital tool in gastric cancer research (67). By precisely controlling parameters such as voltage and solution flow rate, nanofibers with diameters ranging from 200 to 800 nm can be fabricated (68). This dimensional range closely mirrors that of collagen fibrils found in the native mammary extracellular matrix, thereby providing a highly biomimetic model system for studying cancer cell migration behavior and drug tolerance (69, 70). Gastric cancer studies have consistently shown that extracellular matrix remodeling driven by cancer-associated fibroblasts can markedly enhance invasion, and this effect is tightly coupled to matrix composition, organization, and mechanics (71, 72). In peritoneal dissemination, this structural dependence becomes even more explicit. Detached gastric cancer cells must first adhere to and clear the peritoneal mesothelium, and then expand beneath the basement membrane. Adhesion programs centered on integrins and CD44 are widely implicated as key molecular drivers of this sequence (73).

Fiber alignment in electrospun scaffolds should not be viewed as a purely aesthetic morphological parameter. It is an engineering handle to recreate or actively modulate migration tracks *in vitro*. Aligned fibers can amplify directional motility and prolong invasive persistence. This feature is useful for modeling invasion, but it warrants caution when designing anti-dissemination barriers, because overly linearized topographical cues may unintentionally provide residual cells with “escape routes.” Beyond structural guidance, electrospinning also shows promise for solid-state stabilization and activity preservation of monoclonal antibodies (74). When integrated with functional polymers such as hyaluronic acid, electrospun platforms can support localized delivery of antibody therapeutics to tumor sites, reduce systemic exposure, and enable alternative locoregional strategies for agents such as trastuzumab (75).

### 2.3 Nanoparticle loading strategies

In gastric cancer locoregional therapy, the quality of a loading strategy should no longer be ranked by drug payload. It should be judged against three measurable endpoints: sustained and effective intraperitoneal exposure, microenvironment-triggered potentiation, and reduced systemic toxicity.

Mohseni and colleagues developed a coordination-driven epigenetic drug self-assembled nanofiber probe that reprograms tumor cells and achieves superior antitumor efficacy. These nanofibers were generated by simple ultrasonic mixing of a clinically used HDAC inhibitor, SAHA, a DNMT inhibitor, 5-Aza, and zinc ions. The resulting assembly exerts anti–gastric cancer activity through a coordinated mechanism, combining histone hyperacetylation with DNA demethylation (76). More recently, this evaluation logic has been translated into reproducible therapeutic evidence in peritoneal metastasis models. Li and colleagues engineered an electrospun nanofiber membrane, PTX/GSNO@HKUST-1/PLEA, in an advanced gastric cancer peritoneal metastasis setting. The membrane enables continuous intraperitoneal release of paclitaxel and exploits the acidic, glutathione-enriched microenvironment to trigger copper redox cycling, promoting hydroxyl radical generation. In parallel, it releases nitric oxide from GSNO to relieve hypoxia and reduce HIF-1α signaling (77). This design effectively upgrades intraperitoneal chemotherapy into a localized multimodal unit that integrates chemotherapy, chemodynamic killing, and hypoxia modulation. In murine peritoneal metastasis models, the membrane suppressed peritoneal tumor burden, reduced ascites, alleviated cachexia, and prolonged survival, with improved safety. Together, these results provide a direct and mechanistically grounded evidence chain for electrospun membranes as potentiators of intraperitoneal therapy. When the cargo shifts from small molecules to complex therapeutics, the advantage of electrospinning still comes from engineerable architecture, not from simply “being able to load.” On one hand, MOFs and nanoparticles can be stably integrated into fibrous networks and function as local reactors or switches that convert microenvironmental cues into amplified efficacy. The HKUST-1–triggered cascade described above represents a prototypical design. Because HKUST-1 is copper-based, both efficacy and safety depend on Cu(II) leaching kinetics in physiological media, and copper homeostasis pathways are likely to shape cellular exposure under repeated dosing. Accordingly, translational evaluation should quantify time-resolved Cu burden in peritoneal fluid and major organs to assess long-term accumulation/clearance alongside therapeutic benefit.

MOFs are not intrinsically inert; their biological profile is strongly influenced by metal-node and linker chemistry, particle size, and degradation kinetics, which collectively determine the extent and rate of metal-ion release and downstream oxidative or inflammatory signaling. Copper-based MOFs can be particularly effective for redox-enabled therapies, yet uncontrolled Cu ion release and ROS stress may raise off-target toxicity concerns, underscoring the need for careful exposure control and safety testing (78). For intraperitoneal deployment, local safety is additionally constrained by the vulnerability of the peritoneal mesothelium, where injury can promote inflammation and subsequent adhesion/fibrosis; therefore, peritoneal tolerance should be explicitly evaluated rather than inferred from systemic administration alone. Electrospun MOF-based intraperitoneal systems should explicitly address biocompatibility and peritoneal tolerance, which are jointly shaped by MOF composition, surface chemistry, and how freely particles can disseminate within the cavity. In practice, selecting frameworks built from more biogenic metal nodes and biodegradable linkers can reduce concerns associated with metal, while surface functionalization or polymer coatings can attenuate nonspecific bio-interactions and tune degradation and ion-release kinetics (79). In addition, integrating MOFs into electrospun matrices or hydrogels can physically confine the particles, limiting free-particle spread and smoothing burst exposure in the peritoneal space (80). Consistent with these design considerations, studies are increasingly expected to report peritoneal safety readouts—such as mesothelial integrity, local inflammatory cytokine profiles, adhesion, peritoneal-fluid metal concentrations, and tolerability under repeated dosing—together with systemic biochemistry and organ histology to contextualize local–systemic trade-offs.

On the other hand, the main bottleneck for nucleic acid therapies is the duration of effective exposure, rather than a transient peak. Pinese and colleagues delivered siRNA/MSN-PEI using electrospun scaffolds and demonstrated long-term siRNA availability (81). Surface adsorption maintained siRNA accessibility for more than one month, whereas intrafiber encapsulation enabled sustained release for at least five months. Importantly, this approach supported local siRNA retention *in vivo* and produced quantifiable target gene silencing, providing a delivery mode that better matches the “continuous seeding–continuous immunosuppression” trajectory of peritoneal metastasis.

Beyond nucleic acids, fragile cargos such as extracellular vesicles and proteins typically require gentle loading, stable interfacial anchoring, and controlled release. Researchers introduced a polydopamine functional layer onto electrospun PCL nanofibers to immobilize stem cell–derived exosomes and achieve sustained local delivery. Therapeutic benefit was validated in animal models, highlighting electrospun membranes as an enabling engineering backbone for extracellular vesicle–based locoregional therapies (82).

### 2.4 Scale-up, reproducibility, and quality attributes

In the translational pathway of gastric cancer locoregional therapy, the major barrier for electrospinning is not conceptual feasibility. It is the ability to demonstrate batch-to-batch consistency, predict clinical performance, and control risk. Many drug-loaded electrospun systems can yield “effective prototypes” in the laboratory, yet clinical deployment requires a manufacturing-grade quality framework that extends beyond morphology to reproducibility and process robustness (**Supplementary Table 1**).

Accordingly, electrospinning must be governed by systematic quality control paradigms such as quality-by-design, which define and manage critical quality attributes. These attributes include fiber diameter and distribution, porosity, alignment, basis weight and thickness, drug loading and uniformity, residual solvent, mechanical strength, and structural integrity under physiological fluids (83). Collectively, they dictate intraperitoneal retention, release and degradation kinetics, as well as barrier stability and safety at peri-anastomotic sites. Omer and colleagues noted that conventional single-needle electrospinning typically delivers only ~0.01–1 g/h, and this low throughput—combined with high sensitivity to solution state and ambient conditions—creates a recurrent source of scale-up variability and “same formulation, different outcomes.” (84) This challenge is particularly acute in gastric cancer. Peritoneal metastasis control relies on sustained, effective intraperitoneal exposure. Even subtle drift in fiber diameter distribution, pore connectivity, basis weight, or drug uniformity can translate into underexposure with regrowth, or overexposure that increases peritoneal irritation and adhesion risk (85).

To ensure that these CQAs can be reproduced reliably, they must be systematically linked to critical process parameters and critical material attributes. A defined design space should replace empirical trial-and-error, enabling controlled batch-to-batch consistency. As electrospinning moves toward scale-up, free-surface and needleless configurations have been shown to markedly increase throughput and mitigate defects arising from electric-field nonuniformity. For example, Mohandoss and colleagues optimized a production-scale free-surface electrospinning platform, achieving a throughput of 13 g/h and maintaining coefficients of variation below 10% for mat mass and drug content even at high basis weights (86). These results provide a quantitative foundation for high-throughput, quality-controlled manufacturing. In parallel, scalable routes for multi-polymer and composite electrospinning have been systematically summarized, outlining practical boundary conditions for translating laboratory formulations into industrial processes.

**Figure 2:**
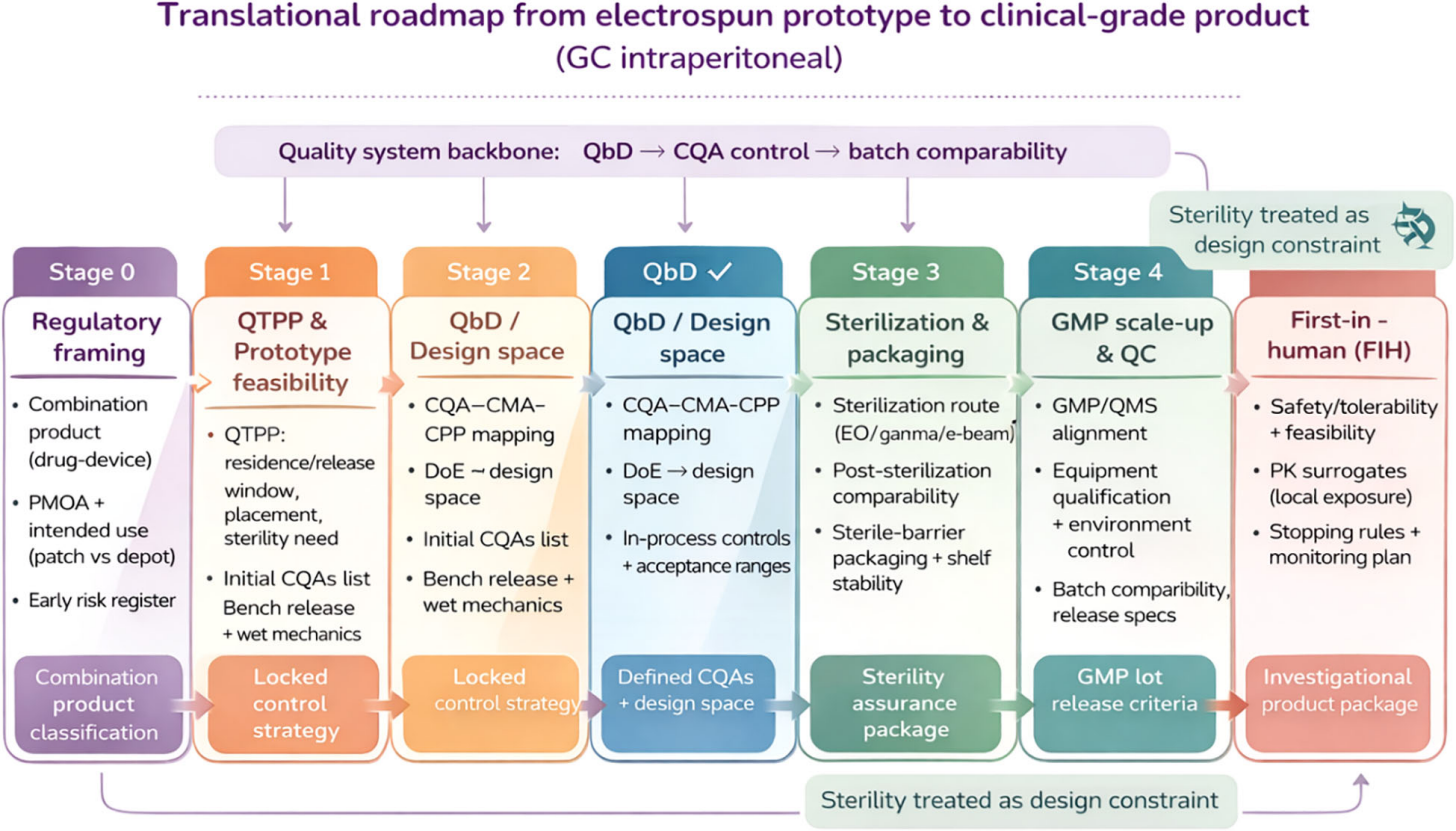
Translational roadmap from electrospun prototype to clinical-grade product for gastric cancer intraperitoneal applications. Stage-gated pathway summarizing regulatory framing as a drug–device combination product, QTPP definition and prototype feasibility, QbD-driven design space establishment (CQA–CMA–CPP mapping and in-process controls), sterilization and sterile-barrier packaging with post-sterilization comparability, GMP scale-up and QC with batch comparability/release criteria, and progression to first-in-human studies focused on safety/tolerability, feasibility, and local exposure surrogates.

From a translational standpoint, drug-loaded electrospun constructs for intraperitoneal use are often positioned as combination products, making early clarification of the primary mode of action (PMOA) and the corresponding CMC/quality expectations a practical first step. Scale-up should be treated as a batch comparability problem under a compliant quality system, and is best operationalized through a QbD lifecycle framework that links QTPP to CQAs/CMAs/CPPs, defines a design space, and locks performance via a risk-based control strategy. Because intraperitoneal products are typically expected to be sterile, sterilization and sterile-barrier packaging need to be incorporated early and validated under recognized standards, with bioburden characterization (ISO 11737), packaging validation (ISO 11607), and—where EO is used—residual control (ISO 10993-7) forming a coherent sterility assurance package (87, 88). Finally, a stage-gated development roadmap—feasibility/QTPP definition, process characterization and design-space establishment, pilot GMP manufacture with stability and sterility assurance, and regulatory submission enabling first-in-human studies—provides a realistic way to align timelines with risk control and evidence generation (**Figure 2**).

Therefore, bringing electrospun materials for gastric cancer locoregional therapy into the clinic requires a shift from “structural novelty” to “manufacturable consistency.” QbD frameworks, supported by DoE-driven process mapping, should be used to stabilize quality control, secure reproducibility, and define an operable therapeutic window. In this context, CQAs become the language of efficacy, and QbD becomes the mechanism to lock performance within a clinically acceptable range.

## 3 Electrospun nanofibers for gastric cancer therapy

The “hard value” of electrospinning in gastric cancer locoregional therapy is not simply loading drugs into fibers. It is encoding local pharmacokinetics into architecture (**Table 2**).

**Table 2:** Translationally relevant electrospun nanofiber use-cases across the GC pipeline.

Application	Design objective	Typical materials	Functional add-ons	Key readouts	Example refs
Resection-bed patch	Sustained local chemo	PCL/PLGA/PLA	Coaxial or multilayer; adhesion tuning	Local recurrence; wound safety	(89)
Intraperitoneal depot	Prolong exposure; reduce adhesions	PCL/PLGA + hydrogel blends	Barrier coatings; staged release	Ascites; peritoneal tumor burden	(90, 91)
Stimuli-responsive mesh	On-demand release	pH/redox-sensitive polymers	Photothermal NIR agents; enzyme-cleavable linkers	Triggered release; synergy	(92, 93)
3D scaffold model	ECM-mimetic invasion/drug gradients	PCL/gelatin; collagen blends	Alignment/stiffness tuning	Invasion metrics; transcriptomics	(94)
CTC capture chip	Enrich rare cells	Nanofiber-coated substrates	Antibody/aptamer functionalization	Capture efficiency; purity	(95)
Biomarker enrichment	Preconcentrate analytes	Nanofiber membranes	Surface chemistry tuning	LOD; recovery; reproducibility	(96)

### 3.1 Advanced electrospinning technologies and controlled drug-release strategies

Coaxial and Janus designs decouple hydrophobic and hydrophilic cargos from protective and functional layers. Multilayer and gradient constructs separate the therapeutic timeline into phases of onset, maintenance, and termination. Porous fibers further reshape diffusion pathways and surface area, enabling more predictable release kinetics. Across drug delivery, tissue engineering, and oncology, a clear consensus has emerged: greater architectural complexity can translate into tighter release control and an engineerable efficacy window, thereby supporting sequential regimens and microenvironment-responsive potentiation.

Time-programmed release is also directly actionable for postoperative recurrence control. Li and colleagues developed a PLGA electrospun membrane, DOX-MPs/ASANF, that hard-wires “rapid killing” and “sustained anti-dissemination” into two release phases (97). DOX microparticles released approximately 58.9% within 24 h to eliminate residual tumor cells. Aspirin released approximately 59.6% over 120 h to continuously suppress platelet activation and platelet–tumor interactions (98). This design significantly reduced recurrence and metastasis in postoperative models. For resection-bed management in gastric cancer, the transferable rule is clear: a fast-releasing cytotoxic component plus a slow-releasing anti-inflammatory or anti-migratory modulator, matched to the perioperative high-risk window rather than a single burst dose.

Layered, sandwich-like architectures further translate the concept of “immune priming first, chemotherapy consolidation later” into an implantable engineering module. Kai Li and colleagues reported a dual-drug depot with a sandwich configuration, in which a 3D-printed outer scaffold delivers the STING agonist MSA-2, while an inner electrospun fibrous layer carries doxorubicin (99). By combining a physical barrier with solubility-driven partitioning, the system achieves sequential release and a cascaded immuno–chemotherapy synergy. Notably, it sustained immune activation and improved suppression of postoperative recurrence and metastasis. The implication for gastric cancer is straightforward. When systemic regimens are constrained by toxicity and adherence, electrospun layered depots can operationalize a reproducible locoregional unit that couples immune reprogramming with downstream cytotoxic consolidation. This strategy is particularly suited for the resection bed in high-risk patients, where controlling microscopic residual disease is the primary objective.

For gastric cancer peritoneal metastasis, the evaluation axis of structural design should align directly with two endpoints: sustained intraperitoneal exposure and microenvironment-triggered sensitization. In murine peritoneal metastasis models, the membrane suppressed peritoneal tumor burden, reduced ascites, extended survival, and showed low toxicity. These results define a practical rule for gastric cancer PM. Retention and stable long-term release should come first. Only then should validated tumor-microenvironment triggers—such as acidity, glutathione, or hypoxia—be layered in to potentiate intraperitoneal chemotherapy. Maximizing drug payload alone is not the objective.

### 3.2 Electrospun nanofibers for locoregional therapy in gastric cancer

#### 3.2.1 Perioperative locoregional delivery: resection-bed tumor control with dual antibacterial and pro-healing functions

Locoregional relapse after curative gastrectomy is not only a matter of “incomplete eradication” of residual tumor cells. A frequent failure route is reactivation of microscopic foci under surgical stress and wound-healing inflammation. Electrospun nanofibrous membranes address this vulnerability by integrating high local drug exposure with spatial confinement into an implantable perioperative intervention unit. Such patches can achieve drug levels at the resection bed that are difficult to reach by intravenous dosing, while minimizing futile diffusion and systemic toxicity.

Recent gastrointestinal tumor studies provide transferable experimental support for this concept. Oxaliplatin-loaded electrospun nanofiber patches have shown sustained locoregional tumor suppression in gastrointestinal cancer models, supporting the feasibility of a “resection-bed chemotherapy depot” with reproducible efficacy (100). Mechanistically, platelet activation and coagulation-linked inflammation during the perioperative window are not bystanders. Platelets can promote invasive programs and metastatic potential through TGF-β signaling and contact-dependent cues. Therefore, high-quality resection-bed designs should not focus on release profiles alone. They should pursue a dual objective: rapid clearance of microscopic residual disease together with suppression of pro-metastatic signals during early repair (101). This strategy aims to match the local exposure window to the highest-risk postoperative phase.

Perioperative complications impose an additional requirement on locoregional delivery systems: they must support antimicrobial control and tissue repair. Otherwise, the material itself can become a risk amplifier. Clinical evidence is unambiguous that anastomotic leakage and related complications significantly worsen long-term outcomes in gastric cancer. More importantly, infection is not only a short-term setback. It can mechanistically promote metastasis. In gastric cancer cohorts and experimental models, postoperative intraperitoneal infection has been shown to induce neutrophil extracellular trap formation and facilitate metastatic progression, coupled to TGF-β-associated invasive programs. These data argue that perioperative biomaterials should be evaluated beyond “drug release.” The relevant clinical metric is a composite function: stable adhesion, antibacterial and anti-inflammatory activity, and pro-healing remodeling. At the anastomotic site, the goal is to avoid increasing leakage risk while lowering infection-driven recurrence potential.

Engineering precedents support this direction. A bilayer electrospun membrane reported in Journal of separation science used stratified functions to jointly manage leakage control and postoperative adhesion risk, illustrating the feasibility of multi-endpoint coordination in surgical settings. In parallel, direct animal evidence shows that nanofabric reinforcement can mechanically stabilize intestinal anastomoses, reducing failure risk and improving local integrity (102). Together, these studies support the feasibility of electrospun patches as reinforcement-oriented perioperative adjuncts, while emphasizing the need to balance reinforcement with anti-adhesion and local safety. For perioperative gastric cancer management, actionable material rules converge on a single principle: a locoregional patch may be engineered to combine a chemotherapy depot with a repair-facing reinforcement interface and/or a peritoneal-facing anti-adhesion barrier, depending on the clinical objective (103).

Perioperative electrospun patches can be engineered either as a peritoneal-facing anti-adhesion barrier or as a tissue-facing, bioresorbable reinforcement interface, and the intended role should be stated because it determines both benefit and risk. The barrier mode prioritizes a smooth, low-fouling surface that minimizes fibrotic tethering without compromising anastomotic healing, whereas the reinforcement mode uses a porous, compliant structure to provide transient mechanical support and pro-healing remodeling within a controlled resorption window. These functions can be integrated in a layered design to spatially separate drug exposure and tissue interaction. Safety concerns mainly arise from excessive persistence, stiffness mismatch, sharp edges, or uncontrolled local drug dose, which may drive foreign-body inflammation, fibrosis, impaired healing, and rare delayed erosion. Therefore, translational designs should emphasize wet-state compliance, atraumatic geometry, predictable degradation, repair-side drug shielding, and report local endpoints including inflammation, adhesion, anastomotic integrity, and delayed erosion.

On the resection-bed side, the priority is high exposure and rapid clearance. Designs should favor an early effective peak followed by sustained release over days to weeks. On the repair-facing side, the priority is wet-state mechanics together with antimicrobial and pro-healing performance. Tissue-friendly natural–synthetic composites are preferred, and layered architectures should be used to shield regenerating tissue from drug-associated irritation.

In the peritoneal setting, an anti-adhesion boundary condition is also required. Bilayer electrospun membranes have been classically validated for adhesion prevention, offering a directly transferable structural logic for postoperative intraperitoneal management in gastric cancer.

#### 3.2.2 Intraperitoneal locoregional therapy: peritoneal metastasis and sensitization of intraperitoneal chemotherapy

Peritoneal metastasis is a major weak point of systemic therapy in gastric cancer. The central limitation is not a lack of drugs, but the inability to achieve sufficient, sustained, and homogeneous exposure within the peritoneal cavity. The plasma–peritoneal barrier restricts drug entry after intravenous administration, leading to inadequate coverage of peritoneal lesions. This has motivated repeated intraperitoneal dosing and pressurized intraperitoneal delivery to increase local concentration and accessibility, with acceptable safety in clinical practice.

However, peritoneal metastasis typically involves diffuse seeding accompanied by a fibrotic stromal context. Even when intraperitoneal drug levels are increased, therapeutic benefit remains constrained by tumor adaptation and tolerance programs shaped by the peritoneal microenvironment. More importantly, peritoneal metastasis follows a continuous cascade that can be mechanistically dissected. After detachment, tumor cells must survive hypoxia, acidosis, and nutrient deprivation in the peritoneal cavity, and then complete adhesion, invasion, and colonization. Hypoxia is not merely a contextual feature. It is an active driver of tolerance. A study in *Redox Biology* provided direct evidence that hypoxia activates HIF-1α and induces the long non-coding RNA PMAN, which promotes cytoplasmic translocation of ELAVL1 and suppresses ferroptosis, ultimately enhancing gastric cancer peritoneal dissemination (104). This indicates that increasing chemotherapeutic exposure alone cannot bypass the survival advantage conferred by redox buffering and ferroptosis resistance.

In parallel, paclitaxel, a cornerstone of intraperitoneal therapy, also faces microenvironment-imposed resistance. Recent work suggests that peritoneal fluid–derived exosomal miR-493 reduces paclitaxel sensitivity by downregulating MAD2L1, further supporting the notion that treatment failure in peritoneal metastasis is often governed by microenvironment-shaped resistance networks (105). Therefore, the engineering focus should shift from “adding more treatment modalities” to “sensitizing key tolerance nodes.” Intraperitoneal strategies should be reorganized around pathological axes such as hypoxia, redox control, and ferroptosis resistance.

Under this framework, the value of electrospun nanofibers is no longer to serve as a generic local sustained-release membrane. It is to package effective intraperitoneal exposure together with microenvironmental sensitization into a single programmable therapeutic unit. A study reported an electrospun membrane that provides sustained intraperitoneal delivery of paclitaxel while incorporating GSNO@HKUST-1 to trigger a cascaded Fenton-like process that amplifies hydroxyl radical generation and releases nitric oxide (106). The nitric oxide component further alleviates hypoxia and reduces HIF-1α signaling. Mechanistically, this design targets two dominant bottlenecks in peritoneal metastasis: diffusion-limited underexposure and hypoxia-driven tolerance.

In murine gastric cancer peritoneal metastasis models, the system reduced tumor burden, decreased ascites, and prolonged survival, offering an animal-validated roadmap for “locoregional chemotherapy plus mechanism-guided sensitization.” These results support a clinically oriented design rule. Electrospun membranes for peritoneal metastasis should prioritize intraperitoneal retention and durable effective exposure, and they should treat hypoxia and redox-buffering tolerance as mandatory sensitization targets rather than optional add-ons.

#### 3.2.3 Anti-adhesion design and sustained intraperitoneal exposure

Postoperative intraperitoneal management in gastric cancer is often underestimated. It requires a barrier to reduce adhesions, yet it also benefits from locoregional materials that can remain in the peritoneal cavity and deliver drugs in a stable manner (90). For anti-adhesion electrospun membranes, the key is not isolation alone. The goal is to suppress the inflammation–oxidative stress–fibrosis cascade, thereby upgrading a “physical barrier” into a “biologically active anti-adhesion” interface.

Qiu and colleagues fabricated an mPEG-b-PLGA electrospun membrane enabling sustained release of ginsenoside Rg3 (107). In animal models of intra-abdominal adhesion, the membrane reduced adhesion scores and adhesion area. These effects were accompanied by downregulation of IL-1, IL-6, and oxidative stress–related mediators, providing direct evidence that electrospun barriers can enhance anti-adhesion efficacy through mechanism-guided pharmacological modulation (108). Oxidative stress is a key pathogenic driver across multiple disease contexts, including cancer, chronic wounds, myocardial infarction, and postoperative adhesions. Under oxidative stress, a rapid surge in reactive oxygen species can cause substantial cellular injury. This has motivated the development of ROS-responsive electrospun nanofibers that either scavenge ROS or enable on-demand drug release at pathological sites (109).

ROS-responsive nanofibers are typically obtained by incorporating ROS-sensitive polymers into the spinning solution. These polymers often contain oxidizable motifs in the backbone or side chains, such as thioethers or ketone and aldehyde groups. In ROS-rich microenvironments, these moieties undergo bond cleavage or oxidation-driven hydrophilization, which in turn triggers drug release. A representative example was reported by Zhang and colleagues. They synthesized a thioether-containing ROS-responsive polyester and electrospun it into a nanofibrous membrane loaded with curcumin and celecoxib to prevent postoperative intra-abdominal adhesion. Oxidation of thioether groups into more hydrophilic sulfoxide or sulfone structures accelerated drug release and simultaneously reduced local oxidative stress, enabling inflammation-linked, microenvironment-driven therapy (109).

A more advanced engineering direction is to integrate anti-adhesion protection with sustained locoregional therapy into a single structured system. The outer layer can be designed as a low-adhesion, anti-fibrotic interface, whereas the inner layer serves as a drug reservoir with tunable release kinetics (110). When needed, a degradable “space-occupying” layer can be incorporated to maintain a local gap and reduce the probability of organ-to-organ contact. In this setting, evaluation metrics must extend beyond short-term adhesion scores.

A central translational issue is that anti-adhesion protection and sustained intraperitoneal drug exposure operate on different biological timelines. Adhesion initiation is dominated by the early postoperative inflammatory, while mesothelial repair is typically completed within 5 days and reperitonealization continues over 7 days; irreversible adhesion remodeling is often discussed in the 7–14 day window. Accordingly, the anti-adhesion layer should prioritize anti-fouling, low-inflammatory surface chemistry and maintain barrier integrity through the first postoperative week, then resorb to minimize persistent foreign-body–driven inflammation or late adhesiogenic risk. In contrast, the chemotherapy-depot layer can be engineered for a longer residence and release window, which has been achieved in multiple intraperitoneal delivery designs (91). Polymer selection can therefore be framed by a “two-window” rule: barrier-facing materials favor hydrophilic and faster-resorbing matrices, whereas depot-facing materials favor slower-degrading polyesters to stabilize mechanical integrity and sustain release; layered or core–shell architectures decouple these requirements in a single construct.

They should include material residence time, degradation trajectory, and whether the release window aligns with the postoperative risk period. Otherwise, clinical benefit is difficult to predict. A practical design rule is therefore a dual-function architecture: a low-adhesion, anti-inflammatory and anti-fibrotic surface coupled to a mechanically stable, sustained-release interior. Barrier integrity over the early postoperative healing window should be treated as a hard requirement for anti-adhesion performance, whereas the drug-depot residence or release window can be independently tuned to match the intended intraperitoneal exposure profile.

#### 3.2.4 Stimuli-responsive systems and locoregional immunomodulation

In gastric cancer locoregional therapy, the upper limit is often not set by the intensity of cytotoxic killing, but by whether local damage can be translated into durable immune control. Stimuli-responsive electrospun systems address this gap by coupling drug release to the tumor microenvironment or the perioperative window (92, 93). This enables higher spatial precision and more sustained temporal coverage.

Magnetic- and temperature-responsive electrospun nanofibers represent one of the most widely used classes of “on-demand release” carriers. Their shared advantage is the ability to convert microenvironmental cues or external stimuli into controllable release behaviors (111). Magnetic responsiveness is typically achieved by incorporating superparamagnetic nanoparticles to enable magnetothermal heating, with ferrites and iron oxides being common choices. Veres and colleagues fabricated electrospun poly fibers loaded with magnetic iron oxide nanoparticles and observed a temperature increase exceeding 8 °C within 5 min under an alternating magnetic field, supporting their potential for magnetothermal-assisted tumor therapy (112).

Temperature-responsive systems usually rely on thermosensitive polymers with a lower critical solution temperature, most prominently poly(N-iso propylacrylamide) (PNIPAAm) (113). Because PNIPAAm fibers can readily disperse in aqueous environments, researchers copolymerized NIPAAm with a crosslinkable monomer, HMAAm, followed by thermal curing to generate nanofibers exhibiting rapid and reversible volumetric transitions during temperature cycling. When loaded with dextran, these fibers displayed a clear “on–off” release profile: repeated heating cycles liberated most of the payload, whereas cooling induced minimal release, enabling precise temperature-gated delivery.

Locoregional biomaterials are best viewed as programmable immunomodulatory interfaces. By gating release with tumor-specific cues such as acidity, glutathione, or ROS, and by implementing “priming-then-consolidation” sequences, electrospun systems can couple cytotoxicity with immune activation and prolong local control into the postoperative high-risk interval. Combination designs that integrate chemotherapy with antiplatelet modulation further suggest that disrupting surgery-induced pro-metastatic signaling is a tractable route for locoregional immunotherapy.

Current evidence is largely based on small-animal models, which may overestimate efficacy relative to human peritoneal physiology and treatment schedules. Safety uncertainties remain for reactive designs, including MOF metal-ion release, ROS amplification, and NO donors, which may cause off-target oxidative, peritoneal irritation, fibrosis, or impaired healing under repeated dosing. Given biodegradation variability and time-dependent drift in release, translational claims should be supported by repeated-dose and longer-term readouts together with manufacturability controls.

## 4 Electrospun scaffolds for biomimetic gastric cancer models

Drug resistance, invasion, and peritoneal dissemination in gastric cancer cannot be fully explained by two-dimensional culture. These phenotypes emerge from a three-dimensional context shaped by spatial architecture, diffusion gradients, and extracellular-matrix cues. Model choice therefore directly determines the clinical credibility of mechanistic conclusions (94).

**Figure 3:**
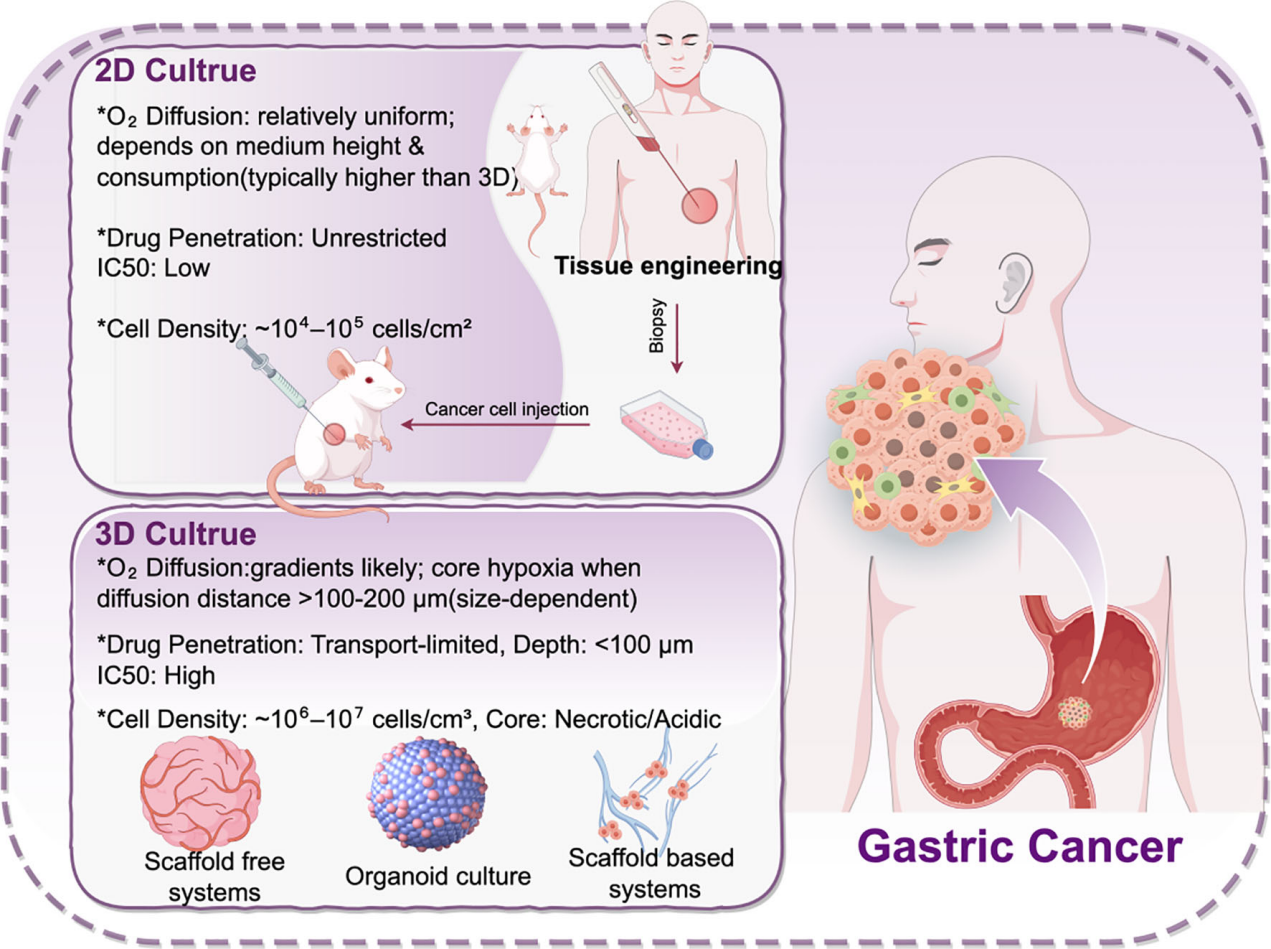
2D versus 3D gastric cancer models and key mass-transport constraints. Schematic comparison of 2D monolayer culture and 3D systems with approximate annotations for oxygen diffusion, drug transport, and representative cell densities. 2D cultures show relatively uniform exposure, whereas 3D constructs exhibit gradients with core hypoxia typically arising when diffusion distances exceed ~100–200 μm and transport-limited drug penetration contributing to higher apparent IC50.

Two-dimensional cultures support rapid screening and tractable mechanistic assays but do not faithfully reproduce the gastric tumor microenvironment. *In vivo* models incorporate systemic pharmacology and host-level constraints that shape therapeutic response. Three-dimensional platforms, including spheroids, organoids, and scaffold-based biomimetic systems, better capture spatial architecture and microenvironmental cues (114). Scaffold-based models further allow programmable control of ECM-mimetic signals through material composition and microstructural design (115), enabling more clinically relevant readouts for drug-response profiling, invasion phenotyping, and microenvironment-focused mechanistic studies (**Figure 3**).

### 4.1 3D growth, hypoxia, and pH microenvironments in phenotypic regulation

The clinically relevant biology of gastric cancer does not unfold on a flat plastic dish. It emerges within a three-dimensional microenvironment shaped by physical barriers, nutrient and oxygen diffusion gradients, metabolic acidosis, and immunosuppressive signaling. As a consequence, 2D monolayer cell lines show two recurrent biases: they tend to overestimate drug sensitivity and underestimate invasive capacity and the evolution of treatment tolerance (116).

PH-responsive systems are particularly well matched to disease-associated acid–base fluctuations (117). They are commonly built by introducing or immobilizing weak polyelectrolytes on electrospun fibers, including alginate, sodium carboxymethyl cellulose, poly(L-lysine), and poly(acrylic acid). Among these, poly(acrylic acid) is widely used because of its simple chemistry, pronounced responsiveness, and low cost. In a representative study, Miranda-Calderon and colleagues fabricated antibiotic-loaded electrospun nanofibers using methacrylic copolymers with distinct dissolution thresholds, including Eudragit L100-55, Eudragit S100, and the pH-independent Eudragit RS100 (118). This design enabled wound dressings to modulate release kinetics in response to pH changes across different healing stages. Beyond weak polyelectrolytes, acid-labile linkers provide an additional route to pH-triggered activation, including imine bonds, CDM linkages, and phenylboronic ester chemistry. For example, Chen and colleagues grafted camptothecin and α-tocopheryl succinate onto hyaluronic acid, and then connected the hyaluronic acid conjugate to poly(lactic acid) through an acid-sensitive CDM linker to generate short nanofibers. Under mildly acidic tumor conditions, CDM cleavage released the hyaluronic acid conjugate, which self-assembled into drug-loaded micelles (119). This mechanism promoted tumor enrichment while reducing the systemic toxicity of chemotherapy. A key advantage of electrospun nanofibrous scaffolds is that their fiber scale and pore architecture can approximate the native extracellular matrix. This provides an *in vitro* 3D framework that remains controllable yet better reflects *in vivo* constraints. As a result, electrospun scaffolds are well suited for postoperative relapse–risk assessment, screening of locoregional delivery strategies, and mechanistic dissection of drug tolerance.

Early gastric cancer–focused evidence has shown that 3D electrospun models can shift pharmacodynamic readouts. Kim and colleagues established a three-dimensional gastric cancer culture on PHBV/collagen peptide electrospun scaffolds and evaluated chemosensitivity (120). Compared with 2D culture, higher drug concentrations were required to achieve comparable cytotoxic effects in the 3D scaffold condition. The authors attributed this divergence to an “architectural barrier” imposed by the 3D microstructure, consistent with diffusion limitations, cell–matrix interactions, and stress adaptation collectively increasing the apparent tolerance threshold. Clinically, the implication is direct: when the goal is to explain resistance and recurrence, 3D electrospun scaffolds capture treatment-pressured cellular responses more faithfully than 2D monolayers (120). More broadly, biomimicry cannot stop at “three-dimensionality.” Decisive microenvironmental variables such as hypoxia and acidosis must be incorporated, otherwise immune escape and relapse cannot be mechanistically explained. In 2025, Xiang and colleagues developed a microfluidic tumor-on-chip platform to recapitulate a hypoxic gastric cancer niche. They showed that hypoxia upregulated FOXO3a and promoted PD-L1 expression, thereby driving resistance to immunotherapy. In a syngeneic mouse model, FOXO3a deficiency restored therapeutic sensitivity by increasing immune-cell infiltration. Consistently, FOXO3a levels in clinical specimens correlated with immunotherapy outcomes (121).

These results suggest a clear direction for model engineering. When electrospun scaffolds are coupled with microfluidic gradient control, the system can move beyond a culture tool. It can help pinpoint actionable tolerance nodes and support a translational workflow that links mechanism discovery to drug screening and, ultimately, patient stratification.

### 4.2 Alignment-guided invasion and mechanobiology

Gastric cancer invasion and peritoneal dissemination are rarely a purely stochastic process (122). They are more consistent with a persistent advance along “migration tracks” defined by stromal fiber alignment, pore connectivity, and stiffness gradients. Fibrotic remodeling can convert a diffuse extracellular matrix into directionally biased linear paths. Tumor cells then translate these structural cues into polarized, sustained migration through integrin-based adhesion and cytoskeletal tension, ultimately shaping invasion depth (123, 124).

Electrospun scaffolds are well suited to interrogate this logic because they allow independent control of fiber organization (random versus aligned), pore architecture, and mechanical range within the same material system. This enables invasion phenotypes in gastric cancer to be moved from correlative descriptions to causal dissection. Under matched drug exposure, differences in invasion often arise first from mechanotransduction of topological and mechanical cues, rather than from changes in proliferation alone. A recent ECM-mimetic electrospinning study provided unusually clear evidence that structure can drive migration. Li and colleagues fabricated biomimetic fiber matrices with distinct architectures and showed that aligned nanofibers markedly increased directional migration and migratory persistence of tumor cells (125). Mechanistically, Cav-1–positive cells on aligned fibers displayed enhanced β1 integrin endocytosis, actin polymerization, stress-fiber assembly, and focal-adhesion remodeling, accompanied by increased YAP activity. Cav-1 inhibition, in turn, weakened this invasive phenotype by disrupting YAP-dependent mechanotransduction (70). Importantly, this Cav-1/β1 integrin trafficking axis, linking adhesion–cytoskeleton reprogramming to YAP activation, is consistent with prior cell-biological evidence implicating Cav-1–mediated β1 integrin internalization in matrix turnover and cell motility. It therefore moves the concept of “alignment promotes invasion” beyond a descriptive observation and frames it as an actionable, mechanistically tractable pathway (125).

Notably, these topography-driven migration effects intersect with EMT plasticity. Aligned or confinement-like fibrous architectures can promote EMT-like invasive behaviors by strengthening integrin-mediated adhesion dynamics and actomyosin tension, thereby favoring polarized, traction-driven migration. Conversely, EMT reprogramming can heighten responsiveness to contact guidance, making directional migration along aligned fibers more persistent. In this view, electrospun matrices provide a tractable platform to test how ECM topography and EMT programs co-modulate invasion, and whether disrupting adhesion–cytoskeleton coupling attenuates EMT-associated migration under defined architectures.

In patients with gastric cancer serosal invasion, collagen remodeling itself can serve as a structured cue of peritoneal metastasis risk. Chen and colleagues quantified collagen features at the serosa-invasive front using multiphoton imaging and developed a collagen-based signature and nomogram to predict postoperative peritoneal metastasis. Their data further suggested that increased collagen density and radial alignment can facilitate invasion (126).

Electrospun scaffolds offer a controllable system to convert stromal architecture into testable, causal determinants of invasion. Defined tuning of alignment and stiffness enables quantitative invasion phenotyping while linking topological cues to Cav-1/YAP-dependent mechanotransduction. This is particularly relevant for peritoneal dissemination–prone disease, where fibrotic tracks may undermine locoregional efficacy and where mechanobiology-informed material design could improve therapeutic robustness.

### 4.3 Patient-derived platforms: organoids and tumor-on-a-chip systems

A clinically useful biomimetic model must directly confront one of the hardest realities in gastric cancer: profound heterogeneity and highly individualized therapeutic responses (**Supplementary Table 1**). Tumor-on-chip systems further introduce dynamic gradients and multicellular interactions, moving the model from static culture toward an interpretable and predictive decision framework.

The value of integrating these platforms is modular and complementary. Organoids anchor biological authenticity, electrospinning reconstructs the structural microenvironment, and microfluidics imposes physiological constraints over time. Together, they enable *in vitro* testing that better approximates resistance evolution and response stratification under clinically relevant selective pressures. A key limitation of conventional PDO culture is that it is often epithelium-dominant and under-represents stromal determinants of response, particularly CAFs. Emerging evidence suggests that CAF–tumor crosstalk can reshape drug sensitivity and mediate chemoresistance, implying that epithelial-only PDOs may overestimate efficacy when stromal resistance dominates. Therefore, tumor–stroma reconstitution combined with electrospun ECM analogues and microfluidics can provide a more clinically aligned framework to interrogate resistance. Another translational variable is genetic drift and clonal selection during extended passaging, which can erode subclonal heterogeneity and shift drug-response phenotypes over time. Practically, this motivates early-passage biobanking, capping passage number for screening, and periodic molecular QC to maintain concordance with the parental tumor before drawing clinically actionable conclusions.

At the level of resources and evidence generation, Yan and colleagues established a systematic gastric cancer organoid biobank spanning normal mucosa, atypical hyperplasia, primary tumors, and lymph-node metastases (127). Integrated whole-exome and transcriptomic profiling showed that this platform captures subtype heterogeneity and subclonal architecture. More importantly for translation, large-scale drug screening uncovered actionable sensitivity signals to agents that are approved or in clinical development, including napabucasin, abemaciclib, and the ATR inhibitor VE-822 (128). These results position patient-derived organoids not merely as passive replicas of inter-patient variation, but as a screening engine capable of generating treatment-guiding hypotheses.

Clinically aligned evidence is also accumulating rapidly. Zhao and colleagues established 57 patient-derived organoids from 73 gastric cancer cases, achieving an approximate success rate of 78% (129). These PDOs preserved key histopathological features of the parental tumors and exhibited markedly heterogeneous responses to standard chemotherapies. Transcriptomic analyses further identified expression signatures associated with sensitivity to 5-fluorouracil or oxaliplatin. Importantly, the predicted responses were validated with 91.7% concordance in PDOX models and matched clinical outcomes, supporting PDOs as a potential front-loaded tool to inform perioperative chemotherapy selection and enable stratified decision-making beyond empiricism (130).

When PDOs are integrated into tumor-on-chip systems, dynamic hypoxia can be modeled as a causal driver rather than a background condition. Xiang et al. identified a hypoxia–FOXO3a–PD-L1 axis that limits immunotherapy efficacy, with FOXO3a loss restoring sensitivity *in vivo* and FOXO3a levels tracking clinical outcomes. This illustrates how chip-enabled control of gradients can expose actionable tolerance nodes in gastric cancer.

While PDO–electrospun–chip integration improves physiological relevance, important determinants of clinical response remain incompletely captured, including CAF-rich stroma, immune components, vascular/perfusion effects, and patient-specific inflammatory cues. Extended passaging can introduce clonal selection and genetic, potentially shifting drug-response phenotypes and weakening clinical concordance if passage number and molecular QC are not controlled. Transport realism is another limitation: diffusion and binding in 3D matrices and chips may still differ from *in vivo* implants, and gradient design can bias outcomes unless standardized and benchmarked. To strengthen interpretability, studies should report passage number limits, periodic genomic, explicit inclusion/exclusion of CAF, and quantitative transport descriptors. Standardization and cross-lab reproducibility—media, matrix mechanics, and assay endpoints—also remain key challenges for translating model-derived “decision rules” into clinically reliable guidance.

## 5 Nanofiber-enabled diagnostics and monitoring

To enable clinical translation of locoregional therapy in gastric cancer, treatment must be coupled to reproducible and real-time tools for response assessment and early relapse warning. Compared with flat substrates and single-marker immunoassays, electrospun nanofibers provide an engineerable interface with a high surface area, a tunable porous network, and facile functionalization. These features support enrichment of low-abundance targets from complex biofluids such as blood and ascites. Nanofiber membranes can also be integrated with electrochemical or optical readouts to build a clinically oriented “enrichment–recognition–quantification” workflow for longitudinal monitoring.

### 5.1 Circulating tumor cell capture substrates

Circulating tumor cells (CTCs) are a key liquid-biopsy readout for recurrence risk assessment and longitudinal response monitoring in gastric cancer. Their clinical deployment is limited primarily by enrichment: CTCs occur at ultra-low frequencies and span heterogeneous phenotypic states, such that affinity-only capture often trades recovery for background contamination (131). As emphasized by Pantel and Alix-Panabières, rare-event statistics and enrichment bias represent the dominant technical constraints, and performance must be demonstrated in unprocessed patient blood rather than under idealized spike-in conditions (132).

Electrospun nanofiber substrates offer a distinct advantage beyond ligand immobilization. Their ECM-mimetic nanotopography and high specific surface area increase productive cell–substrate contacts, enabling physical amplification of binding probability. Zhang and colleagues deposited electrospun TiO_2_ nanofibers onto solid supports and functionalized the surface with anti-EpCAM, achieving improved CTC capture from patient samples that included gastric cancer (133). This study illustrates that nanoscale topological interactions can synergize with immunoaffinity recognition to enhance recovery in complex biospecimens, supporting electrospun interfaces as a viable enrichment material for gastric cancer CTC analysis.

Current CTC technologies increasingly prioritize coverage and downstream usability over absolute yield (134). In practice, robust operation under high-throughput blood flow requires joint control of nanotopography, wettability, and antifouling chemistry to suppress nonspecific adhesion while maintaining recovery (135, 136). Aptamer-based capture further enables reversible binding and gentle release, which is critical for single-cell molecular profiling and functional assays (95). Shen (137) and colleagues demonstrated mild recovery of captured CTCs using aptamer-functionalized nanostructured substrates, and Wang et al. reported in Theranostics a transparent, biocompatible nanostructured surface with aptamer recognition that supported clinically actionable detection and stratification in patient cohorts (138).

A key limitation of EpCAM-centric designs is EMT-associated EpCAM downregulation, which can lead to systematic undercounting of clinically relevant subpopulations (139, 140). Gorges and colleagues showed that EpCAM-based enrichment misses EMT-related CTC subsets, indicating that single-marker strategies do not faithfully represent the full gastric cancer CTC spectrum (141). A practical design rule is to use nanotopography as the physical base layer, add multi-marker or aptamer-based recognition to expand phenotypic coverage, and preserve a low-damage release route to enable downstream analysis and improve clinical concordance.

### 5.2 Biomarker enrichment membranes and biosensor integration

The same nanotopography-enabled enrichment principles that improve CTC recovery can be repurposed as upstream concentration modules for a broader range of low-abundance biomarkers, enabling integrated sensing workflows for longitudinal gastric cancer monitoring (96).

Beyond CTC enumeration, gastric cancer surveillance increasingly relies on longitudinal tracking of multiple low-abundance signals, including circulating proteins, nucleic acids, extracellular vesicles, and inflammatory mediators. Here, electrospun nanofiber membranes serve as more than passive supports: their high surface area and tunable porous networks can be engineered as enrichment layers that concentrate targets upstream of sensing, improving effective mass transfer and signal-to-noise in complex biospecimens (142). Yet enrichment can also amplify background; specificity is often constrained by nonspecific adsorption, cross-reactivity, and phenotypic/epitope heterogeneity in clinical matrices.

Accordingly, enrichment layers are best paired with immunoaffinity recognition and dual-recognition formats. For soluble proteins, sandwich assay designs—capture on the nanofiber surface plus a second detection binder—provide orthogonal recognition that reduces matrix-driven false signals while supporting electrochemical/optical readout. Nanofiber interfaces also allow high probe density and short diffusion distances, enabling integrated “enrich–detect–quantify” workflows compatible with clinical handling (143).

Integration with microfluidics further improves translational readiness by standardizing flow, washing, and signal readout within a closed system, reducing operator dependence and improving reproducibility. This also supports specificity via controlled incubation and stringency washes; for vesicle/cell targets, marker panels can reduce misclassification when single markers overlap. For gastric cancer follow-up, flexible and wearable formats are clinically attractive given the frequency and duration of monitoring (144, 145).

Importantly, objective is not an isolated ultra-low analytical limit, but reproducible performance in real-world follow-up settings. A practical architecture pairs a nanofiber enrichment membrane with a dedicated conductive readout layer and prioritizes antifouling stability, re-calibration capability, and packaging consistency. These engineering controls are essential to convert analytical sensitivity into clinically reliable longitudinal monitoring.

### 5.3 Comparative positioning versus current intraperitoneal standards

Existing intraperitoneal strategies provide useful benchmarks for translational positioning. HIPEC maximizes intraoperative drug exposure under hyperthermia, but it is typically delivered as a single-session bolus and its benefit can be sensitive to procedural heterogeneity and patient selection. PIPAC may improve distribution through aerosolization and can be repeated, yet it requires repeat laparoscopic procedures and faces practical constraints on dosing and scheduling. Repeated intraperitoneal chemotherapy via catheter supports multi-cycle exposure but introduces device-related complications such as obstruction and infection, alongside added patient-burden considerations. Injectable hydrogels can prolong local residence with minimally invasive administration; however, achieving controlled spatial coverage, limiting burst release, and maintaining uniform exposure across diffuse peritoneal implants remain challenging. Within this landscape, electrospun systems are best positioned as implantable, architecture-programmable constructs that enable more deliberate control of residence and release while accommodating microenvironment-responsive potentiation and perioperative barrier-facing requirements, although their evidence base is still largely preclinical and will require stronger safety, manufacturability, and clinical-pathway validation to support translation.

## 6 Materials innovation and functionalization strategies

Electrospun nanofibers are predominantly polymer-based. To date, more than one hundred polymers have been used to construct nanofibrous networks, broadly classified into natural and synthetic materials.

### 6.1 Performance and applications of natural polymers and their composites

Natural polymers, such as collagen, gelatin, silk fibroin, chitosan, and hyaluronic acid, offer excellent biocompatibility and biodegradability. They are readily recognized by cell-surface receptors, which supports cell adhesion and proliferation and makes them attractive for tissue repair and related biomedical applications. By contrast, synthetic polymers including poly(ϵ-caprolactone), poly(lactic acid), poly(vinyl alcohol), poly(ethylene oxide), and polyurethane typically exhibit more reliable spinnability and provide tunable mechanical and physicochemical properties. Notably, many single-component natural polymers remain challenging to electrospin or show insufficient wet-state mechanical stability (146). In practice, blending natural and synthetic polymers is therefore widely used to integrate bioactivity with structural robustness in electrospun fibrous systems (147).

Evidence that aligns with the practical needs of gastrointestinal surgery increasingly supports a “layered composite with multi-endpoint control” strategy. A Biomaterials preclinical study developed a bilayer electrospun membrane with an alginate–gelatin inner layer and a PCL outer layer, and spatially separated mitomycin C and thrombin to integrate sealing adhesion, hemostasis, tissue repair, and anti-adhesion functions within one implant (148, 149). *In vivo*, the patch covered the wound surface and reduced both leakage risk and postoperative adhesions (150). The clinical implication is straightforward for gastric cancer surgery: management around the anastomotic site often requires a single material that stabilizes healing while maintaining a temporary barrier. Bilayer composite electrospun membranes provide a form factor that better matches this real-world requirement (151).

Alginate is attractive because it enables mild ionic crosslinking and shows good biocompatibility. However, neat alginate is difficult to electrospin into stable fibers and the resulting structures are prone to wet-state collapse. A systematic Materials review summarized the common engineering solution: blending with carrier polymers such as PEO or PVA to increase chain entanglement, followed by crosslinking and composite design to tune mechanical integrity and water uptake (152). This workflow converts “spinnability” into clinically relevant membrane performance. Chitosan offers a cationic hemostatic and antibacterial backbone, but practical deployment requires wet-state reinforcement. Experimental studies consistently indicate that electrospun chitosan membranes need physical or chemical crosslinking to improve aqueous stability and mechanical strength (153). With appropriate stabilization, chitosan-based mats can be extended toward surgery-relevant functions including hemostasis, infection control, pro-angiogenic support, and immunomodulation.

Gelatin and collagen excel in cell adhesion and ECM mimicry. Their intrinsic drawbacks are equally clear: rapid swelling and loss of structural fidelity in physiological fluids (154). As a result, gelatin/collagen electrospun systems typically rely on crosslinking or blending with hydrophobic backbones such as PCL to preserve architecture under wet conditions.

Overall, translational deployment in perioperative gastric cancer care and intraperitoneal settings benefits from clear functional partitioning: synthetic components provide wet-state structural reliability, natural components define the bioactive interface for adhesion and healing, and barrier persistence is programmed through layering and crosslinking to match the anastomotic protection window while minimizing premature softening or collapse.

### 6.2 Expanding functional materials and surface engineering strategies

**Figure 4:**
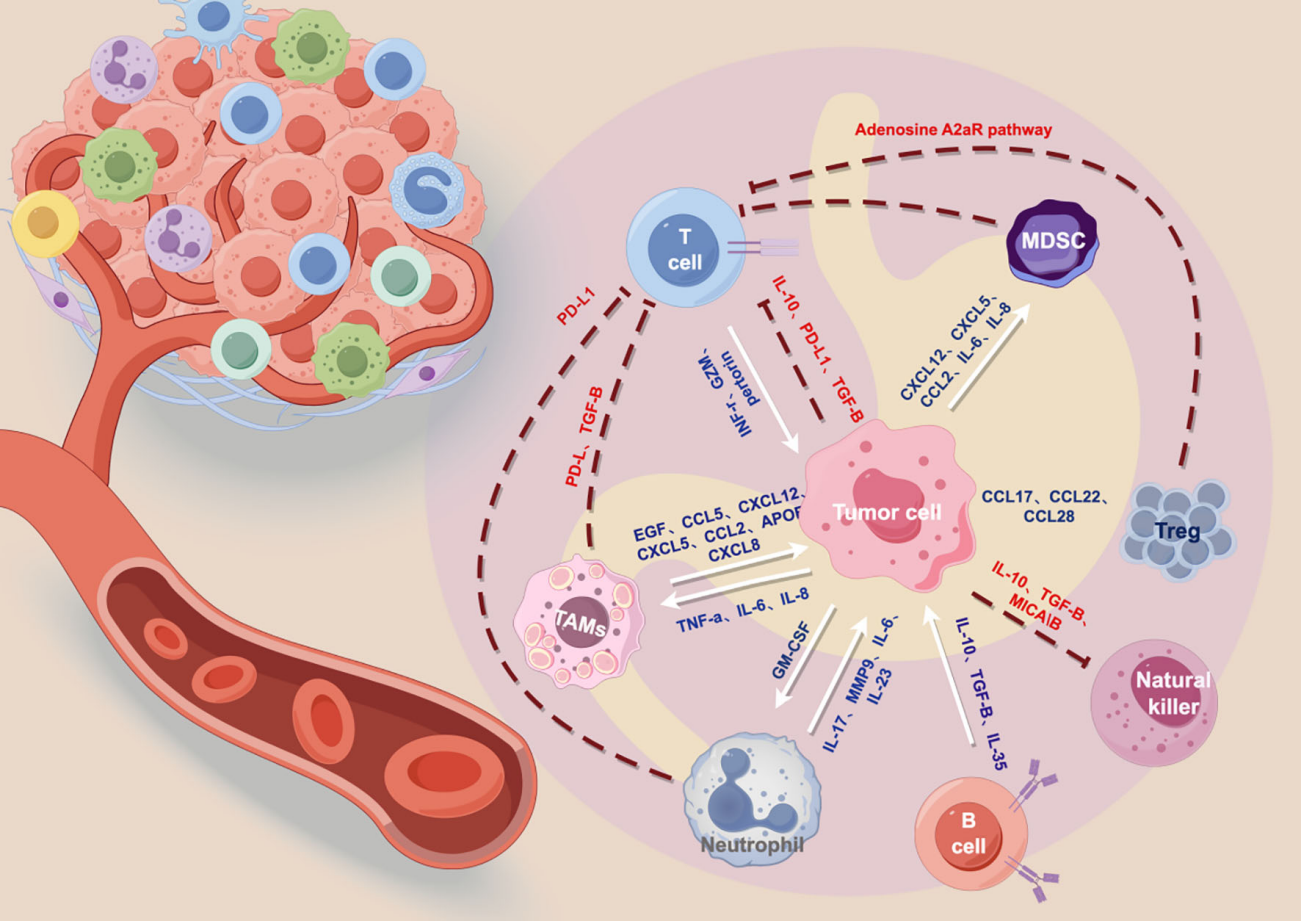
Immune crosstalk and immunosuppressive circuitry in the gastric cancer tumor microenvironment. Schematic overview of key interactions between gastric cancer cells and immune populations, including tumor-associated macrophages, neutrophils, myeloid-derived suppressor cells, regulatory T cells, effector T cells, natural killer cells, and B cells. Tumor cells recruit immunosuppressive subsets through chemokines and inflammatory cues, while dampening antitumor immunity via checkpoint signaling and soluble suppressive mediators. Metabolic inhibitory pathways, exemplified by adenosine-centered circuits, further reinforce immune evasion and therapeutic tolerance.

To achieve durable benefit in gastric cancer, locoregional cytotoxicity should be translated into sustained immune control; otherwise, peritoneal microscopic residues may re-seed under an immunosuppressive milieu. The gastric cancer tumor microenvironment is shaped by coordinated immune evasion, where tumor cells recruit suppressive populations (TAMs, MDSCs, and Tregs) and dampen effector T-cell and NK-cell function through checkpoint signaling, soluble mediators, and metabolic inhibitory circuits such as adenosine (155–159) (**Supplementary Table 1**). This network perspective shifts the goal of locoregional biomaterials from drug loading to triggerable, durable, and experimentally testable immunomodulation aligned with microenvironmental bottlenecks (**Figure 4**).

Release control and barrier function alone are often insufficient in gastric cancer, where hypoxia, a reducing milieu, and antioxidant programs jointly reinforce tolerance (160). Functional materials and surface engineering therefore aim to convert electrospun nanofibers from passive depots into cooperative local systems: tumor microenvironment–activated modules amplify ROS-mediated damage, while gas- or energy-enabled components mitigate hypoxia and improve local penetration (161); engineered interfaces further provide wet-tissue adhesion, antifouling performance, and targeted enrichment to increase intraperitoneal effective exposure while limiting systemic toxicity. Polydopamine is widely used as a universal interfacial primer because it combines hydrated adhesion with catechol/amine chemistry for secondary functionalization and additional photothermal/redox capability (162). Accordingly, an implementable design rule is to integrate a controlled-release scaffold with acidity/GSH-responsive ROS amplification and a hypoxia-alleviating unit, while leveraging polydopamine-enabled interface chemistry to stabilize retention and support modular functionalization within an explicit safety window (**Table 3**).

**Table 3:** Evidence gaps and recommended study designs for GC-focused nanofiber translation.

Use-case	Main risk	Model priority	Manufacturing focus	Clinical endpoint candidate
Postoperative patch	Local toxicity/poor integration	Orthotopic resection-bed recurrence	Dose uniformity; sterilization	Locoregional RFS; complications
Intraperitoneal depot	Uneven distribution/adhesions	Peritoneal dissemination, immune-competent	Degradation window; anti-adhesion	Ascites control; peritoneal PFS
Local immunomodulation	Off-target immune activation	Syngeneic models + immune profiling	Stability of biologics; burst control	Immune infiltration; response rate
3D scaffold decision tool	Limited clinical concordance	Patient organoids with matched outcomes	Standardized scaffold specs	Predictive accuracy vs RECIST
CTC/biosensing device	Assay variability	Prospective longitudinal cohort	Device reproducibility; QA/QC	Sensitivity/specificity; lead time

## 7 Challenges and future perspectives

Electrospun nanofibers feature a high surface area and an interconnected porous network, allowing external cues to be rapidly converted into material-level responses. They are therefore widely used as building blocks for intelligent delivery systems. By incorporating triggers such as pH, ROS, temperature, light, magnetic fields, or ultrasound, stimulus-responsive electrospun fibers can enable sustained release, on-demand dosing, or localized delivery. These platforms have shown broad promise in wound repair, tissue engineering, anticancer therapy, and drug delivery. However, clear barriers remain before clinical translation.

First, the material composition and release pathways are inherently more complex than those of conventional formulations. Toxicity and the therapeutic window depend on formulation, physicochemical properties, dose, administration route, and patient-to-patient variability, making standardized safety definition challenging. Second, triggering is highly context dependent. Implant depth can attenuate light or magnetic stimulation, and disease-site temperature, pH, and oxidative stress vary substantially across patients, leading to shifts in activation thresholds and release kinetics. Third, layered structural and functional integration increases scale-up difficulty and manufacturing cost, placing batch-to-batch reproducibility and industrial quality control at the center of translational risk.

For gastric cancer, the key advantage of electrospun nanofibers lies not in “making fibers” per se, but in turning locoregional therapy into a programmable system. By tuning fiber diameter, pore connectivity, and layered architectures, electrospun constructs can sustain therapeutically relevant intraperitoneal exposure over extended periods. They can further integrate microenvironment-responsive modules that engage hypoxia and redox stress, thereby improving sensitization in peritoneal metastasis. In the perioperative setting, electrospun patches can be designed to address multiple surgical priorities simultaneously, including eradication of microscopic residual disease at the resection bed, infection control, and tissue repair, with the potential to reduce anastomosis-related complications and postoperative relapse.

Beyond gastric cancer, nanotechnology-enabled therapeutic strategies are being actively explored across gastrointestinal malignancies as part of a broader translational movement toward programmable drug delivery and microenvironment-aware therapy. In multiple GI tumor contexts, multifunctional nanoscale platforms are increasingly designed to improve delivery precision and intratumoral exposure, mitigate or bypass resistance mechanisms through pathway- and stroma-informed interventions, and strengthen locoregional control using stimulus-responsive or combinatorial payload logic; this cross-disease momentum provides a useful translational backdrop for positioning electrospun nanofiber systems in gastric cancer as implantable, architecture-programmable interfaces rather than isolated material curiosities. Recent analyses in gastrointestinal stromal tumors (GIST) further illustrate how nanoscale platforms can integrate targeted delivery with tumor microenvironment modulation and explicit translational design principles to address persistent therapeutic limitations, reinforcing that many of the engineering logics discussed here generalize across GI oncology rather than being confined to gastric cancer alone (163–165).

The main translational bottleneck is not a lack of concepts, but insufficient evidence for predictable efficacy and batch-to-batch reproducibility. Response thresholds can vary with patient-specific microenvironments and implantation depth, while increasing structural complexity raises the bar for scale-up, sterilization robustness, and safety assessment. A more realistic path forward is to adopt quality-by-design frameworks, define efficacy and risk boundaries through critical quality attributes, and establish auditable links from process parameters to structure and release behavior. Coupled with scalable electrospinning strategies and sterilization-aware validation, these steps will be essential to advance electrospun nanofibers from “effective prototypes” to clinically deployable, regulatable platforms for gastric cancer locoregional therapy.
